# The impact of social capital on clinical risk management in nursing: a survey in Iranian public educational hospitals

**DOI:** 10.1002/nop2.141

**Published:** 2018-04-15

**Authors:** Mehdi Jafari, Arefeh Pourtaleb, Rahim Khodayari‐Zarnaq

**Affiliations:** ^1^ Department of Health Services Management School of Health Management and Information Sciences Iran University of Medical Sciences Tehran Iran; ^2^ School of Management and Medical Informatics Iranian Center of Excellence in Health Management Tabriz University of Medical Sciences Tabriz Iran

**Keywords:** clinical risk management, hospital, Iran, social capital

## Abstract

**Aim:**

This study aimed to explore the social capital impact on clinical risk management from nurses’ viewpoints.

**Design:**

This was a cross‐sectional and analytical study conduct in six public educational hospitals affiliated to Tehran University of Medical Sciences (TUMS).

**Method:**

Questionnaires were used as the data collection tool. Data were analysed using descriptive statistics, parametric and non‐parametric tests by SPSS 16 at a significance level of 0.05.

**Results:**

Risk management, social capital and all its three dimensions evaluated in moderate level. It is confirmed that the social capital is one of the factors associated with the improvement of clinical risk management. There was a significant relationship between clinical risk management and social capital. In this respect, hospital managers and decision‐makers could enhance clinical risk management by identifying and increasing different dimensions of social capital which consequently led to have a better patient safety culture in hospitals.

## 1 INTRODUCTION

Nowadays, social capital has turned to be a new prevalent concept of any organizations and is being used by politicians and academic experts. Social capital either has a long intellectual history in the social sciences (Sabatini, 2008) and a multi‐dimensional concept, which has impresses effects in many areas of the society (Majedi & Lahsayi Zadeh, 2006). This concept has become an important issue in public health researches in a way that only in 2002, 50 articles on social capital and health was published. Although at first it seemed that there was no scientific evidence about social capital advantages to health but, there is now impressive empirical evidence that highlight it as a determining factor in health outcomes (Poortinga, 2006).

Several studies have shown that good working relationships in nursing environments are significantly important for both nurses and patient outcomes (Aiken et al., 2011; Laschinger & Leiter, 2006; Siddiqui, 2013; Siu, Laschinger, & Finegan, 2008). A review of literature declared that evidence supports the relationship between teamwork and patient safety (Manser, 2009). Also, results from multivariate regression analysis indicated that there is a significant and positive relation between teamwork culture and patient satisfaction for inpatient care and a significant and negative relationship between bureaucratic culture and patient satisfaction. This study suggests that hospitals or other healthcare organizations should strive to develop a culture emphasizing teamwork not those aspects of bureaucracy which are not crucial factor to assure efficiency and quality of care (Meterko, Mohr, & Young, 2004). Also, recently issues related to patient safety has become a major public concern and it has been discussed worldwide in medical and nursing literature (Gardner, Baker, Norton, & Brown, 2002; Kirwan, Matthews, & Scott, 2013; Tzeng & Yin, 2007). However, in this field medical errors explained an important public health problem and pose a serious threat to patient safety (Grober & Bohnen, 2005). The threats pertaining to patient safety can be categorized into four components in organizations: organizational and management practices, workforce employment, work design and organizational culture (Institute of Medicine Committee on the Work Environment for Nurses and Patient Safety, 2004).

## 2 BACKGROUND

Social capital points to characteristics of social organizations such as networks, norms and social trust which facilitate cooperation and coordination to reach the mutual benefits. The World Bank defined the social capital as a set of norms in social structures that makes people capable to gain desired goals by cooperative action (Amirkhani, 2008). It has been said that it is an emerging organizational concept as a result of the positive organizational behaviour in management and psychology literature. Eventually, Social capital refers to the “total resources and values available within a network of individual and organizational relationships, which emanate from this network” (Nahapiet & Ghoshal, 1998). Nahapiet & Ghoshal describe three dimensions for social capital; Structural dimension refers to the pattern of interpersonal relations in a work or group environment; in other words, it refers to the extent to which group members are in contact with each other. Relational refers to the emotional quality of the relationships between group members and includes elements of trust and mutual interactions. Finally, cognitive dimension refers to the extent to which the group members agree on the nature and working objectives with each other (Nahapiet & Ghoshal, 1998).

The concept of clinical risk management refers to the use of risk management techniques in clinical environments to identify, contain and manage risks related to patient safety (Briner, Kessler, Pfeiffer, Wehner, & Manser, 2010). However, better understanding the risk management perceptions leads to more reporting errors; it means that risk management could play a vital role in the prevention and management of medical errors (Miyake, 2001). To learn and prevent the recurrence of errors, proper safety culture and an organizational commitment would be needed (Moullin, 2002).It has been confirmed that organizational culture is a source for enhancing patient safety practices and an important support to prevent errors and avoid their recurrence in clinical risk management (Sammer, Lykens, Singh, Mains, & Lackan, 2010).

Throckmorton et al. found that the majority of nurses are willing to report errors but the possibility to do in this way depends on perceive about the punitive organizational culture (Throckmorton & Etchegaray, 2007). A systematic review about the hospital nursing environment effect on patient mortality indicates that social and environmental attributes of nursing have had a significant effect on the outcomes of care. (Kazanjian, Green, Wong, & Reid, 2005). Also, it has been declared that social capital in hospitals is an essential factor to make a system safe (Firth‐Cozens, 2004). According to the characteristics of social capital which has been mentioned above, firstly, it is plausible that not only individuals, but also complex organizations, such as hospitals have been affected by social capital concept. Secondly, it is assumed that the social capital in hospitals can be a key factor to make a safe environment for patients and this assumption can be tested. Up until now, based on our knowledge very little studies have been found in the context of social capital impact on clinical risk management, especially in Iran. So, the present study aimed to examine the relationship between social capital and clinical risk management from nurses’ perspective in in public, educational hospitals affiliated to Tehran University of Medical Sciences (TUMS).

## 3 MATERIALS AND METHODS

This was a cross‐sectional, analytical study conducted in 2015. The statistical population consisted of the nurses who have worked in six educational, public hospitals affiliated to Tehran University of Medical Sciences. Four inpatient wards with similar specialties (internal, surgery, gynecological surgery and paediatrics) were selected randomly in each hospital and the sample size of 252 nurses was investigated based on stratified sampling with the confidence level of 95% and an error of less than 5%. Data were collected by using two questionnaires as follow:

### 3.1 Social Capital Questionnaire

The researchers embarked on designing a researcher constructed questionnaire according to the library and electronic studies in the field of social capital and considering the available research and scientific resources, including books, dissertations and retrieved relevant questionnaires with regarding to Nahapiet and Ghoshal's conceptual model. The content and face validity of the questionnaire were assessed through expert opinion. The Cronbach's alpha coefficient of .92 was obtained for the test–retest reliability of the questionnaire. The five‐point Likert scale (strongly agree, agree, moderate, disagree and strongly disagree from (5–1) was applied. This questionnaire includes 31 questions in two parts:

- Demographic and professional profile, including age, sex, work experience, marital status, type of employment and education
- Social capital consisting of structural (11 items), cognitive (6 questions) and relational (14 questions) dimensions. The total score of the questionnaire varied from 31–155 points. We also defined the 0–31 scores as very low, 31–62 low, 62–93 moderate, 93–124 good and 124–155 as excellent for social capital.

### 3.2 Risk Management Questionnaire

The variable, entitled “risk management” was measured by standard 6‐item scale questionnaire. This measure has been translated into English by Ernestman et al. and the Cronbach's alpha coefficient reliability of 0.81 has been reported for it (Ernstmann et al., 2009). This questionnaire was translated into Persian and its validity and reliability have been examined and approved like social capital questionnaire with the Cronbach's alpha coefficient of 0.86. The items of this questionnaire were about determining the very important causes of accidents, taking appropriate measures after the incidence of error‐like situations, reporting nosocomial infections, improving patient safety, promoting quality of services and controlling the events related to treatment in the hospital. Like what mentioned above, the five point Likert scale (strongly agree, agree, moderate, disagree and strongly disagree from (5–1) was applied. The total score of this questionnaire fluctuated from 6–30 points. We described the 0–6 scores as very low, 6–12 low, 12–18 moderate, 18–24 good and 24–30 as excellent for risk management.

### 3.3 Ethical issues

The confidentiality of data obtained from the questionnaire was one of the researcher's obligations. The freedom of respondents to answer a part or whole of the questionnaire was taken into account. All participants provided a verbal informed consent for this study.

Data were analysed using descriptive statistics (frequency percentage, mean and standard deviation), as well as Spearman correlation coefficient test (for assessing the correlation between risk management and social capital, relational and structural dimensions) and Pearson correction test (for assessing the correlation between risk management and cognitive dimension). We also used one way ANOVA, Kruskal‐ wallis and Mann–Whitney test. In correlation test, risk management was considered as the dependent variable while social capital and its dimensions were considered as the independent variables. SPSS 16 software was used for data analysis at significance level of 0.05.

## 4 RESULTS

The total number of participants in this study consisted of 215 nurses (response rate: 85.3%). Among the study population 184 (85.6%) were female and 31 (14.4%) were male. 141 participants (65.6%) were married and 74 (34.4%) were single.

In terms of age, the majority of them 115 (53.5%) were under 30 years old and the mean age for men was (30.32 SD 6.27) and for women (32.63 SD 8.82). 179 (83.3%) participants had bachelor's degree.

Regarding work experience, the number of 98 nurses (45.6%) had less than 5 years of work experience, 62 (28.8%) had work experience between 5–10 years and 27 participants (12.6%) had work experience between 10–15 years. The mean year for work experience among men and women were (7.32 SD 5.75) and (8.42 SD 7.04), respectively. The respondents age range (62–22) was 40 years. Finally, in terms of employment status, most of the nurses 48.8% were contract staff, 21% were official employees, 18.5% of them were contractual and 11.6% were mandatory services. Table 1 summarizes the basic demographic data in study population.

**Table 1:** Demographic characteristic in study population

Variable	*N*	%
Gender		
Male	31	14.4
Female	184	85.6
Age (years)		
Less than 30	115	53.5
31–40	74	34.4
41–50	13	6.0
More than 50	13	6.0
Marital status		
Single	74	34.4
Married	141	65.6
Education		
Diploma or lower	10	6.7
Bachelor	179	83.3
Master or higher	21	10
Work experience (years)		
Less than 5	98	45.6
5–10	62	28.8
10–15	27	12.6
15–20	10	4.7
More than 20	18	8.4
Employment status		
Contracted	105	48.8
Official	45	21
Under contract	40	18.6
Mandatory service	25	11.6

Our findings showed that the mean and standard deviation for social capital in these six hospitals were (79.15 SD 13.27). The maximum possible score obtained for social capital could be155. It means that the participants in this study evaluated social capital in moderate level which needs to be supported and improved for being good. The mean and standard deviation for clinical risk management in these hospitals were (13.80 SD 3.36) while the highest score in this part could be 30. It can be concluded that the risk management here didn't have a good situation and it was nearly low (Table 2). Generally, in this study all three dimensions of social capital were had a same situation and evaluated in a moderate level.

**Table 2:** Mean and standard deviation of social capital and clinical risk management

Dimension	Mean (*SD*)	The highest possible score
Structural	28.15 (5.71)	55
Cognitive	15.56 (4.17)	30
Relational	35.10 (6.99)	70
Social capital	79.15 (13.28)	155
Risk Management	13.82 (3.36)	30

In this study, Spearman correlation coefficient test was used to analyse the correlation and there was a significant relationship between clinical risk management and social capital. Among the dimensions of social capital, the relational dimension was significantly associated with risk management (*p* < .05) and all three dimensions had direct correlation with risk management. In addition, social capital and clinical risk management were correlated (*p* < .05). The positive correlation between these two variables (*r* = .14) suggests that increased social capital slightly boosts risk management and this relationship is significant (*p* < .05) (Table 3).

**Table 3:** The statistical relationship between social capital component and risk management

Research dimension	Risk management	
	*r*_*s*_	*p*‐value
Structural	.104	.131
Cognitive	.042	.539
Relational	.185	.007
Social capital	.142	.040

Among the demographic characteristic variables in participants, marital status had a significant relationship with both social capital and relational dimension of social capital (*p* < .05). Moreover, the single participants had a higher mean value in social capital and also the relational dimension of social capital than married participants (*p* < .05). The other demographic variables were not significantly associated with social capital and risk management (*p* > .05) (Table 4).

**Table 4:** The statistical relationship between demographic characteristic, social component and risk management

Research variable	Structural	Cognitive	Relational	Social capital	Risk management
	*p‐*value				
Age	.385	.315	.161	.097	.510
Gender	.855	.293	.728	.918	.144
Education	.544	.955	.910	.844	.320
Marital status	.058	.877	.006	.039	.097
Job experience	.437	.264	.089	.068	.503
Employment status	.718	.501	.344	.289	.082

## 5 DISCUSSION

This study aimed to investigate the effect of social capital dimensions on clinical risk management in hospitals affiliated to TUMS in 2015. The findings confirmed that higher social capital is associated with increased risk management from the nurses’ perspective. However, we can assume that social capital would enhance trust and open communications between healthcare team members in work environment and, in this way; it can create an atmosphere of common values and shared beliefs. Therefore, social capital can lead to risk management practices improvement such as reporting errors, specification of the causes of accidents and appropriate measures after the incidence of errors. Research about Risk management in radiology departments in hospitals affiliated to Isfahan University of Medical Sciences showed that risk management issue and its related factors were not in good condition (Abdi, Maleki, & Khosravi, 2011). Another study on risk management assessment in Tehran's public educational hospitals declared that it is essential for hospital managers regarding to the position of risk management to improve the quality of healthcare services while creating a safe environment for both staff and patients. Adopting policies and planning for learning and monitoring risk management activities and procedures should be pursued seriously (Zaboli, Karamali, Salem, & Rafati, 2011). These results were consistent with other study conducted in Germany (Ernstmann et al., 2009).

In these six hospitals, from nurses’ viewpoints the risk management level had been evaluated as fairly low which means that from participant's perspectives there is not enough consideration to the patient safety culture issues and improvement approaches. These findings also have been reported in most of the previous studies conducted in Iranian hospitals society (Abdi et al., 2011; Rezapoor, Tanoomand Khoushehmehr, Bayat, Arabloo, & Rezapoor, 2012; Zendegani, Zare zadeh, Montaseri, & Rabei, 2015). So, determining and evaluating the patient safety culture level in hospitals especially public educational ones should be a continuous process. Akbari et al. in their survey on the patient safety culture concluded that the total average of patient safety culture and scores of all its 12 dimensions were lower than the acceptable criteria that urgently enhancement is needed (Akbari, Zarei, Gholami, & Mousavi, 2015). Not only in Iran but in other international countries such as Turkey, Taiwan, USA and Netherlands there is an emphasis to improve patient safety culture (Bodur & Filiz, 2010; Top & Tekingündüz, 2015; Wagner, Smits, Sorra, & Huang, 2013) The European commission for Health and Consumer Protection proposed policy areas for action to improve patient safety in Europe include the following: (a) the establishment of “an effective reporting and learning mechanisms”; (b) the establishment of “redress mechanisms” for fair compensation to injured patients; and (c) the “development and use of knowledge and evidence” (Conklin, Vilamovska, De Vries, & Hatziandreu, 2008).

In the current study from nurses’ viewpoint, social capital and all its three dimensions included structural, cognitive and relational had a moderate status. The findings indicate that more attention is needed to these aspects of organization culture such as trust, shared ethical values and aims. These items would provide social networks and communities and enable them to act cooperatively. This finding is consistent with Ommen's study (Ommen et al., 2009). Here, we can say that nursing leaders would have critical roles in taking certain steps to enhance social capital and, thereby, improve clinical risk management. Organizational behaviours such as managerial support, teamwork, nurse participation in hospital affairs can be used. In other study apparently stated that for building social capital in healthcare organizations nursing leaders can use ecological thinking to build the vital resource of social capital by taking concrete steps to commit the necessary human and material resources (Hofmeyer & Marck, 2008). Oliver Ommen concluded that increasing social capital in hospitals requires strategies for reinforcing a culture of trust and willingness to work together (Ommen et al., 2009).

Firth‐Cozens suggested that the best way to establish trust is to support trustworthiness and trusting, both in leaders and staff. Lower bureaucracy, open communication, support for teamwork, rewards for reporting and for showing trustworthy behaviour, experience of fair results from reporting, empowering staff and human resources and risk management policies to support open and fair culture are some of the organizational structures and processes (Firth‐Cozens, 2004). Other researches confirmed that nurses should be rewarded for identifying and reporting errors (Page, 2004).

Clinical risk management meetings should include all nurses and physicians in an interdisciplinary approach to establish mutual trust and willingness to report errors in all areas of health care. Knowledge about human and systemic factors that lead to clinical errors and risk management strategies for effective management should be an integral part of nursing training (Gregory, Guse, Dick, & Russell, 2007). The increase in social capital in hospitals requires some strategies to strengthen the culture of trust and willingness to cooperate with each other. Management measures in organization should be taken to create a desired working environment through the creation of a tendency to provide mutual support and pursue common values and goals in a hospital.

Results showed that among the demographic characteristic there is a significant relationship between marital status and social capital and the relational dimension. Also, single participants had a higher mean value in the social capital and relational dimension of social capital than married ones. This means that single people achieved more social capital and support than married people in hospitals. Another study conducted in Kermanshah hospitals showed that there was no significant relationship between patient safety culture and gender, employment status, educational level and work classification (*p* > 0/05) (Almasi, Pourmirza, Ahmadi, Godarzi, & Ahmadi, 2015). In Zaboli et al. study statistical difference observed between gender and risk management (Zaboli et al., 2011).

This study had some potential limitations that may affect the results. Some respondents may not express their true opinions in some areas due to fear of negative effect on their work. Using subjective data obtained from questionnaire could have had some bias to measure social capital and risk management. However, for a precise evaluation, we need to conduct our study in a larger scale and among the other personnel working in these hospitals such as physicians and other clinical staff.

### 5.1 Limitation of the study

This study had some potential limitations that may affect the results. Some respondent may have not expressed their true opinions in some areas due to fear of negative effect on their work. In addition, considering the fact that the hospitals here are not the real sample of Iranian society, we should cautiously generalize results of this study to other hospitals in the country.

## 6 CONCLUSION

On the whole, the results of this study confirm that there is a positive correlation between social capital and clinical risk management; therefore, social capital is one of the factors associated with the improvement of risk management. In this respect, hospital managers and other decision‐makers who work in this field can attempt to improve clinical risk management by increasing different dimensions of social capital in hospitals. We suggest more applied and interventional studies for better understanding about the organizational culture, the relation between social capital and clinical performance indicators’ outcome such as clinical risk management and patient safety.

## 7 ETHICS APPROVAL AND CONSENT TO PARTICIPATE

This study was approved by Vice Chancellor for Research and Technology related to Tehran and Iran Universities of Medical Sciences. The confidentiality of data obtained from the questionnaire was one of the researcher's obligations. The freedom of respondents to answer a part or whole of the questionnaire was taken into account. All participants provided a verbal informed consent for this study.

## 8 AVAILABILITY OF DATA AND MATERIAL

The dataset used and/or analysed during the current study is available from the corresponding author on reasonable request.

## ACKNOWLEDGEMENTs

This study was approved by Vice Chancellor for Research and Technology related to Tehran and Iran Universities of Medical Sciences. Authors thank all members involved in the Research Department of universities, directors, officials and honourable staff of public educational hospitals affiliated to Tehran University of medical sciences.

## CONFLICT OF INTEREST

The authors declare that they have no competing interests.

## AUTHORS’ CONTRIBUTIONS

MJ contributed to the conception and design of the study and he has been involved in revising manuscript critically for important intellectual content. AP contributed in drafting the article and revising it critically for important intellectual content. RKZ made substantial contributions to conception and design, analysis and interpretation of data. All authors read and approved the final manuscript.
